# Pharmacological Intensification Strategies in Highly Refractory Obsessive–Compulsive Disorder: Evidence Synthesis and a Tertiary-Care Case Series

**DOI:** 10.3390/jcm15124796

**Published:** 2026-06-20

**Authors:** Mario Pinzi, Alessandro Cuomo, Pietro Carmellini, Claudia Libri, Maria B. Rescalli, Caterina Pierini, Alessia Santangelo, Benjamin Patrizio, Andrea Fagiolini

**Affiliations:** Department of Molecular Medicine, School of Medicine, University of Siena, 53100 Siena, Italy; alessandro.cuomo@unisi.it (A.C.); pietro.carmellini@unisi.it (P.C.); claudia.libri@ao-siena.toscana.it (C.L.); m.rescalli@student.unisi.it (M.B.R.); c.pierini@student.unisi.it (C.P.); a.santangelo7@student.unisi.it (A.S.); benjamin.patrizio@student.unisi.it (B.P.); andrea.fagiolini@unisi.it (A.F.)

**Keywords:** obsessive–compulsive disorder, treatment-resistant OCD, serotonergic augmentation, mirtazapine, topiramate, pharmacological intensification, clozapine-induced OCD, dual SSRI therapy

## Abstract

**Background:** Treatment-resistant obsessive–compulsive disorder (TR-OCD) remains a major therapeutic challenge. Although current guidelines recommend optimized serotonin reuptake inhibitor (SRI) therapy, clomipramine switching, exposure and response prevention, and antipsychotic augmentation, a substantial proportion of patients continue to experience severe and disabling symptoms. In such cases, clinicians may consider pharmacological intensification strategies beyond guideline-endorsed algorithms. **Methods:** This study combines a structured narrative synthesis of pharmacological strategies for TR-OCD with a retrospective observational case series from a tertiary OCD referral clinic. Treatment resistance was defined as failure to achieve at least a 35% reduction in Yale–Brown Obsessive Compulsive Scale (Y-BOCS) score after at least two adequate SRI trials, including clomipramine, and optimized exposure and response prevention when available. Five patients treated with pharmacological intensification strategies were included. The primary outcome was percentage change in Y-BOCS score at 12 weeks. **Results:** The case series illustrates five strategies used in highly refractory OCD: supratherapeutic SSRI dosing, SSRI plus mirtazapine augmentation, dual SSRI therapy, serotonergic intensification in a clozapine-treated patient, and glutamatergic/GABAergic augmentation with topiramate. Baseline Y-BOCS scores ranged from 28 to 32. At 12 weeks, symptom reduction ranged from 23% to 36%. One patient met criteria for response, three showed near-response, and one demonstrated partial improvement. No cases of serotonin toxicity or clinically significant cardiac complications occurred. **Conclusions:** These cases suggest that carefully monitored pharmacological intensification may be feasible in selected specialist settings, but efficacy and safety require confirmation in prospective controlled studies. **Recommendations**: Pharmacological intensification should be reserved for highly refractory patients managed in specialist services, implemented with gradual titration, structured serotonin toxicity and electrocardiographic monitoring, and explicit individualized risk–benefit discussion; dual SSRI therapy should be regarded as the most experimental and highest-risk serotonergic option; and prospective controlled studies incorporating standardized functional outcomes are needed to refine patient-selection criteria and clarify which patients may benefit.

## 1. Introduction

Obsessive–compulsive disorder (OCD) is a chronic and disabling psychiatric condition characterized by intrusive obsessions and repetitive compulsions that cause marked distress and functional impairment. Lifetime prevalence is estimated at approximately 2–3%, and the disorder is frequently associated with substantial psychosocial burden, occupational dysfunction, and elevated suicide risk [1]. In addition to functional impairment, OCD is associated with substantial long-term disability and reduced quality of life, with population studies showing levels of impairment comparable to major depressive disorder and schizophrenia [2]. A recent systematic review and meta-analysis confirms that effective treatment is associated with significant improvements in quality of life, underscoring the functional stakes of treatment resistance [3]. Neurobiological models implicate dysfunction within cortico–striato–thalamo–cortical (CSTC) circuits, with converging evidence from neuroimaging, pharmacological, and neurosurgical studies [4].

Current international treatment guidelines consistently recommend selective serotonin reuptake inhibitors (SSRIs) and cognitive–behavioral therapy with exposure and response prevention (ERP) as first-line interventions [5,6]. Treatment response in OCD differs from that observed in major depressive disorder. Higher SSRI doses and longer treatment duration are frequently required to achieve clinically meaningful improvement, and response often emerges after 10–12 weeks of sustained therapy [7]. Despite optimized pharmacological and psychotherapeutic interventions, a substantial proportion of patients remain symptomatic. It is estimated that up to 40–60% of individuals do not achieve full remission after adequate SSRI trials, and approximately one-third meet operational criteria for treatment-resistant OCD (TR-OCD) [8]. Guideline-supported next steps following SSRI non-response include switching to clomipramine or augmentation with low-dose antipsychotics. Nevertheless, even after implementation of these recommended strategies, a subset of patients continues to experience severe and functionally impairing symptoms. In such cases, clinical decision-making extends beyond strictly guideline-endorsed algorithms. While emerging evidence supports additional approaches—including glutamatergic augmentation and neuromodulation—these strategies remain variably supported and are often reserved for highly specialized settings [9,10]. Recent narrative and systematic reviews of treatment-resistant OCD pharmacotherapy emphasize that the evidence base beyond antipsychotic augmentation remains heterogeneous and largely derived from small randomized trials and observational studies [11].

Within this clinical space, serotonergic combination strategies have been described in smaller studies and specialist practice. In addition to summarizing the available evidence, the present report aims to illustrate real-world clinical decision-making in a tertiary OCD referral setting, highlighting how pharmacological intensification strategies may be selected, implemented, and monitored in highly refractory patients.

To define the clinical gap addressed here more precisely, this manuscript is concerned with specialist-level pharmacological decision-making in highly refractory OCD once optimized SRI/clomipramine therapy and, when available, exposure and response prevention (ERP) have failed. Although the majority of strategies considered act on the serotonergic system, the case series and evidence synthesis deliberately extend to glutamatergic/GABAergic augmentation (topiramate) and contextualize neuromodulation, ketamine, N-acetylcysteine, and memantine, so that the term “pharmacological intensification” is used in its broader sense. Treatment resistance is, moreover, unlikely to be a single homogeneous phenotype: it may vary across symptom dimensions (e.g., checking/doubt, unacceptable aggressive or sexual thoughts, relationship-centered obsessions), comorbidities, level of insight, tic history, and cognitive/metacognitive profile, which provides a rationale for the personalized, case-based approach adopted below.

Throughout this manuscript the following terms are used. Non-response denotes failure to achieve a clinically meaningful change in Yale–Brown Obsessive Compulsive Scale (Y-BOCS) score; partial response denotes a Y-BOCS reduction below 25%; near-response denotes a 25–34% reduction; and response denotes a reduction of at least 35%. Treatment-resistant OCD refers to inadequate response despite at least two adequate SRI trials (including clomipramine) and optimized ERP when available, whereas treatment-refractory OCD denotes persistence of disabling symptoms despite multiple guideline-supported strategies, including antipsychotic augmentation. The 35% threshold for response and the 25–34% band for near-response were adopted a priori in line with widely used operational definitions in the TR-OCD literature, because in highly refractory patients clinically relevant change frequently falls short of conventional response criteria and a sub-threshold improvement may still be functionally meaningful. For consistency, the abbreviation SRI (serotonin reuptake inhibitor) is used throughout to denote the pharmacological class as a whole, including clomipramine, whereas SSRI is reserved for selective serotonin reuptake inhibitors specifically.

## 2. Materials and Methods

### 2.1. Study Design

This study integrates two complementary components:

(1) A structured narrative synthesis of published evidence on pharmacological strategies in treatment-resistant obsessive–compulsive disorder (TR-OCD);
(2) A retrospective observational case series conducted in a tertiary OCD referral clinic.

The objective was not to establish treatment efficacy but to describe real-world implementation, pharmacological considerations, and safety governance when serotonergic combination strategies are used in highly refractory patients.

These five patients were identified consecutively among adults with treatment-resistant OCD managed at the clinic who underwent a pharmacological intensification strategy and completed at least 12 weeks of follow-up during the study period. The clinical characteristics, background pharmacotherapy, intensification strategy, and 12-week outcomes of the five included patients are summarized in Table 1. As the case series is retrospective, a complete screening denominator of all eligible patients was not prospectively recorded and could not be reliably reconstructed; the resulting possibility of selection bias is explicitly acknowledged in Section 6.

The observational component of the study is reported in accordance with the STROBE guideline for observational studies [12], and the five case descriptions follow the CARE reporting guideline for clinical case reports [13].

### 2.2. Literature Search Strategy

The aim was not to perform a systematic review or meta-analysis, but to provide a clinically oriented synthesis of the pharmacological strategies most relevant to treatment-resistant OCD (TR-OCD) in the contemporary literature; accordingly, the literature component is reported as a structured narrative review rather than a systematic review intended to quantify pooled treatment effects.

A structured literature search was conducted in PubMed/MEDLINE and Google Scholar for articles published between January 2015 and December 2025. In PubMed/MEDLINE, terms were combined using Boolean operators: (“obsessive-compulsive disorder” OR “OCD”) AND (“treatment-resistant” OR “treatment-refractory”) AND (“SSRI” OR “serotonin reuptake inhibitor” OR “clomipramine” OR “antipsychotic augmentation” OR “mirtazapine” OR “dual SSRI” OR “topiramate” OR “memantine” OR “N-acetylcysteine” OR “ketamine” OR “deep brain stimulation” OR “transcranial magnetic stimulation”). Google Scholar was searched with the same terms in simplified form, and the first pages of results were screened pragmatically for additional peer-reviewed records not already retrieved.

Eligible sources were peer-reviewed articles published in English that addressed the pharmacological treatment of TR-OCD and reported clinically interpretable outcomes; non-English publications and conference abstracts without full text were excluded. Titles and abstracts were screened for relevance, followed by full-text review when appropriate. Priority was given to randomized controlled trials, meta-analyses, systematic reviews, and high-quality observational studies; case reports and small case series were included selectively when they addressed under-studied or emerging strategies for which controlled evidence was unavailable, and their lower evidentiary weight was made explicit when cited. Seminal studies predating the search interval (e.g., foundational randomized controlled trials and pivotal augmentation studies) were retained through backward citation (reference-list) searching and expert selection, given their continued clinical relevance. Major international OCD practice guidelines were identified through purposive searching and appraised narratively.

Consistent with the structured narrative design, the number of records was not tracked through a formal PRISMA flow diagram and no quantitative risk-of-bias instrument was applied; instead, the methodological strength and limitations of each cited source (e.g., open-label versus controlled design, sample size) are discussed narratively within the text.

### 2.3. Case Series: Clinical Setting, Patient Selection, Definitions and Monitoring

The case series was conducted in a tertiary OCD referral clinic specializing in treatment-resistant presentations. For the purposes of the clinical case series component of this study, treatment-resistant OCD (TR-OCD) was operationally defined as failure to achieve at least a 35% reduction in Yale–Brown Obsessive Compulsive Scale (Y-BOCS) score following at least two adequate trials of serotonin reuptake inhibitors (including clomipramine), each administered at maximally tolerated or guideline-recommended high doses for a minimum of 10–12 weeks, and optimized exposure and response prevention (ERP) therapy when available.

Symptom severity was assessed using the Yale–Brown Obsessive Compulsive Scale (Y-BOCS) [14]. This operational definition was applied solely for patient selection within the retrospective case series. Because adequacy of exposure and response prevention (ERP) is central to a label of treatment resistance, the ERP status of each patient was recorded individually and is reported in Table 2 (i.e., whether ERP was unavailable locally, refused by the patient, incomplete/discontinued, completed but ineffective, or ongoing). Y-BOCS assessments were administered by the treating psychiatrists at the tertiary clinic. Baseline ratings were obtained prospectively at the index visit, whereas the present analysis of week-12 outcomes was conducted retrospectively from the clinical records; ratings were therefore not blinded to treatment, a limitation acknowledged below. To complement the Y-BOCS, the Clinical Global Impression–Improvement and –Severity scales (CGI-I/CGI-S) were used to summarize global change, and the approximate daily time occupied by rituals was documented narratively at each visit.

Patients were included if they:

- met DSM-5 diagnostic criteria for obsessive–compulsive disorder;
- fulfilled the operational definition of TR-OCD described above;
- had undergone multiple prior pharmacological interventions;
- were considered for serotonergic combination strategies after failure of guideline-supported approaches.

Treatment decisions were made within routine clinical care following multidisciplinary discussion and individualized risk–benefit assessment. Five patients treated with pharmacological intensification strategies during the study period were included in the case series. Each case illustrates a distinct pharmacological intensification strategy discussed in the evidence synthesis, including supratherapeutic SSRI dosing, SSRI plus mirtazapine augmentation, dual SSRI therapy, serotonergic intensification in a clozapine-treated patient, and glutamatergic augmentation with topiramate.

The primary outcome was percentage change in Y-BOCS score from baseline to week 12.

Clinical response was defined as a reduction of ≥35% in Y-BOCS score, while reductions between 25% and 34% were considered near-response.

Secondary outcomes included adverse events and electrocardiographic QTc parameters.

### 2.4. Safety Monitoring Procedures

All serotonergic combination strategies were implemented within a structured monitoring framework that included gradual dose titration and avoidance of simultaneous rapid escalation of multiple agents.

Patients were systematically monitored for serotonin toxicity using clinical decision rules informed by the Hunter Serotonin Toxicity Criteria [15], with particular attention to neuromuscular hyperactivity, autonomic instability, and altered mental status. Electrocardiograms were obtained at baseline and during follow-up in patients receiving serotonergic combination regimens. QT intervals were corrected using the Fridericia formula (QTcF). Predefined safety thresholds prompted treatment re-evaluation for QTcF ≥ 480 ms and treatment discontinuation for QTcF ≥ 500 ms or if arrhythmic symptoms occurred. Prior to initiation of combination regimens, concomitant medications were systematically reviewed for potential cytochrome P450-mediated drug–drug interactions. When SSRI–clomipramine combinations were implemented, clomipramine doses were maintained conservatively and titrated gradually with close clinical monitoring. Follow-up assessments were conducted over a minimum period of three months after initiation of combination therapy. Functional improvement and adverse effects were documented during routine clinical visits. The results of this monitoring are reported in Table 2: electrocardiograms obtained at baseline and at week 12 showed no QTcF above the predefined 480 ms threshold and no clinically significant change in any patient. For the clozapine-treated patient (Case 4), measured plasma clozapine and norclozapine concentrations are reported in the corresponding case description and in Table 2.

### 2.5. Ethical Considerations

This retrospective case series used de-identified clinical data collected during routine clinical care. Written informed consent for publication of anonymized clinical information was obtained from all patients included in the case series.

### 2.6. Artificial Intelligence Use Statement

Artificial intelligence-assisted tools were used exclusively for limited linguistic and stylistic refinement of the manuscript. No AI system contributed to the conception, design, literature selection, clinical evaluation, data analysis, interpretation of the findings, or scientific conclusions. All intellectual content, methodological decisions, and responsibility for the integrity and accuracy of the work rest entirely with the authors.

## 3. Evidence Synthesis: Treatment Strategies in Treatment-Resistant OCD

### 3.1. Neurobiological Circuit Model of OCD

Obsessive–compulsive disorder (OCD) is increasingly conceptualized as a disorder involving dysfunction within cortico–striato–thalamo–cortical (CSTC) circuits linking the orbitofrontal cortex, anterior cingulate cortex, striatum, globus pallidus, and thalamus. Converging evidence from neuroimaging, neurophysiological studies, and neurosurgical interventions indicates that abnormal activity within these circuits contributes to the generation and persistence of obsessive–compulsive symptoms [16,17]. Functional imaging studies consistently demonstrate increased activity within orbitofrontal and anterior cingulate regions as well as the caudate nucleus in individuals with OCD, with partial normalization following effective pharmacological or behavioral treatment [18,19]. Multiple neurotransmitter systems modulate CSTC circuit function. Serotonergic signaling plays a central regulatory role in cortical–striatal connectivity and provides the primary rationale for serotonin reuptake inhibitors (SRIs) as first-line pharmacotherapy. Dopaminergic modulation within striatal circuits has been implicated in habit formation and compulsive behavioral reinforcement, supporting the use of antipsychotic augmentation in treatment-resistant cases. Increasing evidence also suggests that glutamatergic dysregulation contributes to abnormal excitatory signaling within CSTC pathways [4]. Recent integrative reviews have consolidated this circuit-based model, explicitly linking serotonergic, dopaminergic and glutamatergic dysfunction within CSTC loops to current and emerging treatments, and contemporary systematic reviews of functional connectivity (fMRI/EEG) confirm distributed large-scale network abnormalities extending beyond classical CSTC regions in OCD [20].

Within this neurobiological framework, pharmacological strategies in treatment-resistant OCD can be conceptualized as attempts to restore functional balance within these interconnected neural circuits.

### 3.2. Guideline-Supported Pharmacological Strategies

Current international treatment guidelines recommend serotonin reuptake inhibitors (SSRIs) and cognitive–behavioral therapy with exposure and response prevention (ERP) as first-line interventions for obsessive–compulsive disorder [5,21]. Compared with major depressive disorder, pharmacological treatment of OCD typically requires higher SSRI doses and longer treatment duration, with clinically meaningful improvement often emerging only after 10–12 weeks of sustained therapy [1,7]. When response to optimized SSRI therapy remains inadequate, recommended next-step strategies include switching to another SRI, switching to clomipramine, or augmentation with low-dose second-generation antipsychotics such as risperidone or aripiprazole. Meta-analytic evidence indicates that antipsychotic augmentation can produce clinically meaningful symptom reduction in approximately 30–40% of patients with SRI-resistant OCD [22,23]. Recent network meta-analyses of randomized controlled trials in SRI-resistant OCD likewise identify antipsychotic augmentation among the most effective next-step options, while underscoring the heterogeneity and limited size of the underlying evidence [24,25]. Despite these guideline-supported strategies, a substantial proportion of patients continue to experience persistent and functionally impairing symptoms. It is commonly estimated that approximately one-third of individuals with OCD meet operational criteria for treatment-resistant illness after multiple adequate pharmacological trials [8].

### 3.3. Pharmacological Strategies Beyond Current Guidelines

In patients with persistent symptoms despite guideline-based treatment, clinicians may explore pharmacological intensification strategies in specialized clinical settings. These approaches are generally supported by smaller clinical studies, observational reports, and expert consensus rather than large randomized trials [26]. One such strategy involves supratherapeutic SSRI dosing. Evidence suggests that SSRIs demonstrate a dose–response relationship in OCD, with greater symptom reduction observed at higher doses compared with lower therapeutic ranges [27]. In a controlled trial involving nonresponders to standard-dose sertraline, escalation to doses between 250 mg and 400 mg daily resulted in greater improvement in obsessive–compulsive symptoms compared with continuation of 200 mg daily [28]. Observational safety data also suggest that higher SRI doses may be tolerated in selected patients when implemented with careful monitoring [29]. Another strategy involves serotonergic combination therapy aimed at enhancing serotonergic modulation in patients who demonstrate partial but incomplete response to monotherapy. Augmentation of SSRIs with mirtazapine has been investigated in exploratory trials and randomized studies, with some evidence suggesting additional improvement in obsessive–compulsive symptoms [30,31]. Other serotonergic combinations—including SSRI–clomipramine regimens or dual SSRI strategies—have been described in clinical reports and small observational series, although robust controlled evidence remains limited and careful safety monitoring is required due to potential pharmacokinetic interactions and serotonergic toxicity [32,33]. In parallel, growing interest has focused on glutamatergic modulation as a potential treatment target in OCD. Agents influencing glutamatergic neurotransmission have been investigated based on evidence of abnormal excitatory signaling within CSTC circuits [4]. Among these agents, topiramate—an anticonvulsant with AMPA/kainate receptor inhibition and GABAergic enhancement—has been evaluated as adjunctive therapy in treatment-resistant OCD. Randomized controlled trials have reported reductions in compulsive symptom severity when topiramate was added to ongoing SSRI treatment [34,35], although tolerability limitations may restrict its broader use. Memantine and N-acetylcysteine (NAC) have been evaluated as adjunctive treatments in treatment-resistant OCD. Randomized controlled studies have reported mixed but sometimes promising results for memantine augmentation of SSRIs, while NAC has demonstrated modest symptom improvement in some controlled trials [36,37]. Recent systematic reviews and meta-analyses of randomized trials have provided updated, cautiously favorable estimates for N-acetylcysteine augmentation and for memantine augmentation in refractory OCD, while emphasizing the small number and heterogeneity of the available trials [38,39]. Rapid-acting glutamatergic interventions such as ketamine have been investigated as potential treatments for refractory OCD. A randomized controlled crossover trial demonstrated rapid reductions in obsessive symptoms following intravenous ketamine administration [40]. Subsequent studies using active-controlled designs have further supported the rapid but often transient anti-obsessional effects of ketamine [41]; a recent systematic review of ketamine in OCD reinforces these signals while highlighting the brief duration of effect [42]. For patients who remain refractory to pharmacological intensification, neuromodulation provides a further option: recent meta-analyses support the efficacy of deep brain stimulation and of deep transcranial magnetic stimulation in treatment-resistant OCD, although these interventions remain confined to highly specialized centers [43,44]. Taken together, these findings suggest that in selected patients with highly treatment-resistant OCD, pharmacological intensification strategies targeting serotonergic and glutamatergic systems may represent potential therapeutic options. However, the available evidence remains limited, and such approaches should generally be implemented within specialist clinical settings with careful monitoring of safety and tolerability. To illustrate how specialist-level serotonergic combination strategies may be implemented and monitored in real-world practice, we present a small case series from a tertiary OCD referral service.

## 4. Case Series

**Figure 1:**
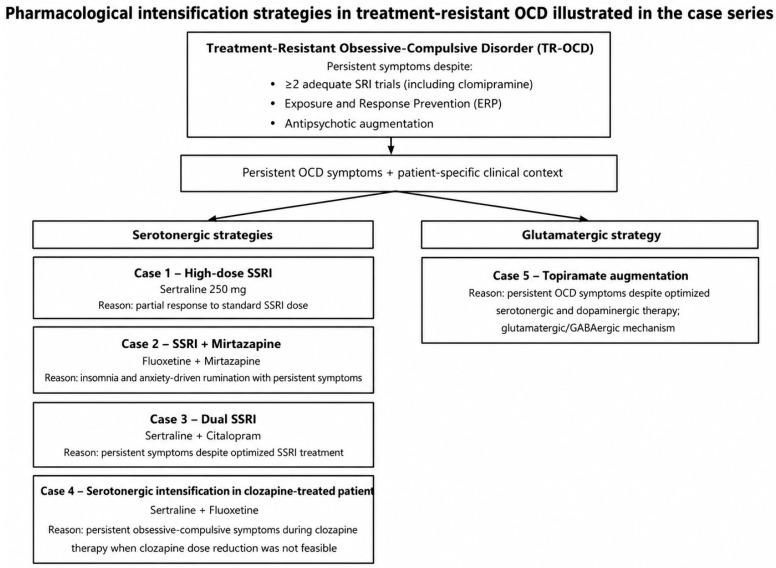
Clinical symptom profiles observed in the present case series and corresponding pharmacological intensification strategies selected to target persistent obsessive–compulsive symptoms.

The following case series illustrates five pharmacological strategies used in specialist management of treatment-resistant OCD, each representing a distinct mechanism of serotonergic or glutamatergic modulation discussed in the preceding evidence synthesis. Before the individual descriptions, the cohort is summarized in aggregate. The series comprised five patients (three men, two women) with a mean age of 47.8 years (range 33–62). All met the operational criteria for TR-OCD, with a heavy prior treatment burden (multiple adequate SRI trials including clomipramine in all cases, and previous anti-psychotic augmentation in four). Dominant symptom dimensions were checking/doubt (Cases 1, 3, 5), relationship-centered obsessions (Case 2), and aggressive/sexual obsessions with schizoaffective comorbidity (Case 4); one patient had a tic history (Case 3). Baseline Y-BOCS scores ranged from 28 to 32 (mean 30.0). At 12 weeks, Y-BOCS reductions ranged from 23% to 36% (mean ≈ 31.8%). Using the predefined thresholds, one patient met the response criterion (≥35%), three were near-responders (25–34%), and one showed partial improvement (<25%). No patient experienced serotonin toxicity, clinically significant QTc prolongation, or major cardiac events, and background psychotropic doses were otherwise held stable during the 12-week observation window. Given that only one of five patients met the predefined response criterion, these outcomes should be read as signals of possible benefit in highly selected patients rather than as evidence that the strategies are effective. Figure 1 illustrates, for each case, the dominant clinical symptom profile and the pharmacological intensification strategy chosen to target persistent obsessive–compulsive symptoms.

### 4.1. Case 1—Supratherapeutic SSRI Dosing in Treatment-Resistant OCD

Presentation. A 62-year-old woman with long-standing OCD (onset in early adulthood, episodic course, ~40 years’ duration) was referred for persistent, disabling symptoms; the current exacerbation had lasted ~1 year. The picture was dominated by doubt-related obsessions with severe checking compulsions (locking doors, switching off appliances), with verification rituals of 3–4 h/day, loss of employment, and preserved insight. Baseline Y-BOCS was 28 and CGI-S 6 (severely ill).

Prior treatment. Adequate trials with limited benefit including fluvoxamine, citalopram, and clomipramine; aripiprazole augmentation discontinued for insufficient benefit. ERP was refused by the patient. At referral she was on sertraline 200 mg/day, with risperidone 2 mg/day and lorazepam 1 mg at bedtime (stable for months); symptoms remained severe despite ongoing antipsychotic augmentation.

Rationale. Given persistent severe symptoms, good tolerability of sertraline 200 mg/day, and limited benefit from antipsychotic augmentation, a cautious escalation beyond the conventional maximum dose was considered; baseline ECG was unremarkable.

Intervention and monitoring. Sertraline was titrated from 200 to 250 mg/day over two weeks, with all concomitant medications unchanged and clinical/ECG monitoring; ECGs at baseline and week 12 were normal, with no QTcF prolongation (<480 ms).

Outcome. Improvement appeared after ~4 weeks at 250 mg, with ritual time falling from 3–4 h to ~1 h/day and greater daily autonomy. At 12 weeks Y-BOCS fell from 28 to 18 (−36%), corresponding to CGI-I 2 (much improved) and meeting the predefined response criterion.

Adverse events. Well tolerated; no gastrointestinal symptoms, agitation, insomnia, tremor, or other features of serotonergic toxicity; cardiovascular parameters and QTc stable.

Follow-up. Sertraline maintained at 250 mg/day without further escalation.

### 4.2. Case 2—SSRI Augmentation with Mirtazapine in Treatment-Resistant OCD

Presentation. A 59-year-old woman with OCD since age 20 (~39 years’ duration) was referred for persistent obsessive doubts and functional impairment. Symptoms were predominantly relationship-centered obsessions and pathological doubt (recurrent doubts about whether her marital relationship was “correct”), with prolonged rumination, reassurance seeking, and several hours/day of mental reviewing, causing distress and reduced occupational functioning. Comorbid generalized anxiety and severe insomnia were present. Baseline Y-BOCS was 29 and CGI-S 6 (severely ill).

Prior treatment. Numerous trials with limited benefit, including clomipramine; aripiprazole discontinued for akathisia and lack of benefit; risperidone stopped for hyperprolactinaemia. ERP was ongoing. At evaluation she was on fluoxetine 60 mg/day (partial improvement, persistent rumination); pregabalin had been added for anxiety, reducing daytime anxiety but with minimal effect on sleep and rumination.

Rationale. Given persistent symptoms with prominent insomnia and anxiety-driven rumination, mirtazapine augmentation was selected for its favorable profile on sleep and anxiety alongside serotonergic modulation.

Intervention and monitoring. Mirtazapine was titrated to 45 mg/day with fluoxetine maintained at 60 mg/day, with clinical monitoring for tolerability and interactions; ECGs at baseline and week 12 were normal, with no QTcF prolongation (<480 ms).

Outcome. Improvement appeared within 4–6 weeks, with better sleep continuity, reduced evening rumination, and decreased intensity of intrusive relational doubts. At 12 weeks Y-BOCS fell from 29 to 19 (−34%), corresponding to CGI-I 2 (much improved) and consistent with near-response.

Adverse events. Generally well tolerated; transient sedation during early mirtazapine titration, diminishing over time; no significant cardiovascular or neurological effects.

Follow-up. Fluoxetine–mirtazapine combination maintained during follow-up.

### 4.3. Case 3—Dual SSRI Strategy in Severe Treatment-Resistant OCD

Presentation. A 47-year-old man with OCD since childhood and a history of motor tics (~35 years’ duration) was admitted for severe, persistent symptoms. The picture was dominated by intrusive doubts about everyday actions and compulsive checking rituals occupying several hours/day, with substantial impairment. Baseline Y-BOCS was 32 and CGI-S 7 (among the most extremely ill).

Prior treatment. Numerous adequate trials, including high-dose fluoxetine (80 mg/day), fluvoxamine (300 mg/day), and clomipramine; aripiprazole (10 mg/day) and risperidone (2 mg/day) without benefit; prior mirtazapine discontinued for limited benefit. ERP was ongoing. At admission he was on fluoxetine 80 mg/day plus gabapentin 400 mg three times daily, with persistent severe symptoms.

Rationale. After comprehensive evaluation (baseline labs and ECG), a monitored dual SSRI strategy was chosen in preference to other options judged less suitable: SSRI–clomipramine was relatively contraindicated by additive serotonergic and cardiac-conduction risk and prior limited tolerance of tricyclics; renewed antipsychotic augmentation had already failed; and memantine, N-acetylcysteine, or neuromodulation were either unavailable within the treatment window or declined. Sertraline and citalopram were chosen in part for their relatively limited CYP450 inhibition.

Intervention and monitoring. Fluoxetine was discontinued and replaced with sertraline 200 mg/day plus citalopram 40 mg/day, titrated gradually under structured monitoring for serotonin toxicity, with serial ECG given citalopram’s dose-related QT liability; ECGs at baseline and week 12 were normal, with no QTcF prolongation (<480 ms).

Outcome. Improvement appeared within 4–6 weeks, with reduced pathological doubt and fewer/shorter checking rituals. At 12 weeks Y-BOCS fell from 32 to 21 (−34%), corresponding to CGI-I 2 (much improved) and consistent with near-response.

Adverse events. Generally well tolerated; no gastrointestinal symptoms, agitation, tremor, or serotonergic toxicity; no relevant QTc prolongation on serial ECG.

Follow-up. Dual SSRI regimen maintained with outpatient follow-up.

### 4.4. Case 4—Serotonergic Intensification for Clozapine-Associated Obsessive–Compulsive Symptoms

Presentation. A 33-year-old man with schizoaffective disorder and comorbid OCD since adolescence (~20 years’ duration) was referred for severe, disabling obsessive–compulsive symptoms, dominated by intrusive aggressive and sexual obsessions (thoughts of harming others; disturbing sexual thoughts involving family members) with mental neutralization rituals occupying several hours/day and precluding employment. Baseline Y-BOCS was 31 and CGI-S 6 (severely ill).

Prior treatment. Adequate prior trials including clomipramine. Maintenance clozapine 100 mg twice daily (200 mg/day) plus valproate 500 mg twice daily, with psychotic symptoms clinically stable but OCS severe. ERP was ongoing. Clozapine reduction was judged unsafe given a documented history of psychotic relapse on previous antipsychotics and on lower clozapine exposure.

Rationale. With clozapine reduction not feasible, obsessive–compulsive symptoms were targeted directly. Sertraline was initiated and titrated to 200 mg/day (clozapine/valproate unchanged) with only partial improvement, so a monitored serotonergic intensification was added; fluoxetine was chosen partly for its relative weight-neutrality, with attention to potential CYP-mediated clozapine accumulation.

Intervention and monitoring. Fluoxetine 40 mg/day was added to sertraline 200 mg/day, with serial ECG and plasma-level monitoring. Psychotic stability was monitored systematically: across 12 weeks there was no re-emergence of delusions, hallucinations, or affective decompensation. The available trough showed clozapine 157 ng/mL and norclozapine 75 ng/mL (below the 200–600 ng/mL reference range), not suggestive of fluoxetine-related accumulation; ECGs at baseline and week 12 were normal, with no QTcF prolongation (<480 ms).

Outcome. Improvement appeared within 4–6 weeks, with reduced intensity and frequency of intrusive aggressive and sexual obsessions and less engagement in neutralization rituals. At 12 weeks Y-BOCS fell from 31 to 21 (−32%), corresponding to CGI-I 2 (much improved) and consistent with near-response.

Adverse events. Generally well tolerated; no serotonergic toxicity; no relevant QTc prolongation; no psychotic relapse.

Follow-up. Combined serotonergic regimen maintained alongside ongoing clozapine therapy.

### 4.5. Case 5—Glutamatergic Augmentation with Topiramate in Treatment-Resistant OCD

Presentation. A 38-year-old man with OCD since adolescence (~25 years’ duration) was referred for persistent, disabling symptoms, predominantly pathological doubt and checking compulsions with prolonged mental rumination, precluding employment. No comorbidity was reported. Baseline Y-BOCS was 30 and CGI-S 6 (severely ill).

Prior treatment. Multiple adequate SRI trials, including clomipramine, and second-generation antipsychotic augmentation, without meaningful benefit. ERP was ongoing. At evaluation he was on a maximally optimized regimen of paroxetine 60 mg/day, vortioxetine 20 mg/day, and brexpiprazole 2 mg/day, with persistent severe symptoms.

Rationale. Given persistence despite optimized serotonergic and dopaminergic agents, topiramate was selected purely on glutamatergic/GABAergic grounds (reducing pathological frontostriatal hyperactivity); the weight-related and binge-eating advantages of topiramate did not apply to this patient, who had no such features.

Intervention and monitoring. Topiramate was added and titrated to 100 mg/day, with the existing regimen unchanged and monitoring for neurological adverse effects, interactions, and ECG when indicated; ECGs at baseline and week 12 were normal, with no QTcF prolongation (<480 ms).

Outcome. Improvement appeared within 4–6 weeks, with reduced obsessive doubts and less time in checking rituals. At 12 weeks Y-BOCS fell from 30 to 23 (−23%), corresponding to CGI-I 3 (minimally improved) and indicating partial response.

Adverse events. Generally well tolerated; mild transient paresthesia during early titration, resolving spontaneously; no significant cognitive or cardiovascular effects.

Follow-up. Topiramate augmentation maintained as part of ongoing treatment.

## 5. Discussion

The present study combines a structured synthesis of the literature with a clinical case series illustrating pharmacological intensification strategies in patients with highly treatment-resistant obsessive–compulsive disorder (TR-OCD). The primary aim of this case series was not to provide evidence of efficacy but to illustrate real-world clinical decision-making in highly refractory cases. All individuals included in the series had persistent and functionally disabling symptoms despite multiple adequate serotonin reuptake inhibitor (SRI) trials, and several had previously undergone augmentation with antipsychotic agents without sustained clinical benefit. Across the five cases, a progressive range of pharmacological intensification strategies was implemented, including supratherapeutic SSRI dosing, serotonergic augmentation with mirtazapine, dual SSRI therapy, serotonergic treatment in the context of clozapine therapy, and glutamatergic augmentation with topiramate. These complex pharmacological regimens reflect the highly refractory nature of the clinical presentations and the multiple prior treatment failures observed in this patient population. The pharmacological strategies described here should not be interpreted as general treatment recommendations. They reflect individualized clinical decision-making in highly refractory cases managed within specialist settings with careful monitoring and detailed risk–benefit evaluation. These observations should therefore be considered hypothesis-generating rather than prescriptive. Despite their heterogeneity, these approaches share a common clinical objective: overcoming partial response to optimized serotonergic pharmacotherapy in patients with persistent symptoms. Treatment-resistant OCD remains a major therapeutic challenge. Even when guideline-recommended treatment algorithms are followed—including high-dose SSRI therapy and exposure and response prevention (ERP)—approximately one-third of patients continue to experience clinically significant symptoms [1,27]. In such cases clinicians frequently explore pharmacological intensification strategies aimed either at enhancing serotonergic modulation or targeting additional neurobiological systems implicated in OCD pathophysiology. The purpose of the present case series is therefore primarily illustrative, highlighting possible clinical strategies and safety considerations in highly refractory cases rather than providing definitive evidence of treatment efficacy.

### 5.1. Serotonergic Optimization and Combination Strategies

The first four cases illustrate different forms of serotonergic pharmacological intensification. In the first case, escalation of sertraline beyond the conventional maximum dose produced clinically meaningful improvement after previous SSRI trials and antipsychotic augmentation had failed. Previous literature also suggests that antipsychotic augmentation may be particularly effective in patients with comorbid tic disorders, potentially reflecting a stronger contribution of dopaminergic dysregulation within cortico–striatal circuits in this subgroup [22,45]. Meta-analytic evidence suggests that SSRIs demonstrate a dose–response relationship in OCD, with greater symptom reduction observed at higher therapeutic doses [7,27]. Controlled trial data also indicate that dose escalation beyond 200 mg/day of sertraline may lead to additional improvement in some non-responders [28]. From a clinical perspective, these observations raise the possibility that in some patients apparent treatment resistance may reflect incomplete rather than absent serotonergic responsiveness, although this hypothesis requires further investigation in controlled studies. The second case illustrates augmentation of SSRI therapy with mirtazapine. Beyond its antidepressant properties, mirtazapine exerts indirect serotonergic modulation through presynaptic α2-adrenergic antagonism and 5-HT2/5-HT3 receptor blockade. This pharmacological profile may be particularly useful when obsessive symptoms are accompanied by insomnia, hyperarousal, or anxiety-driven rumination. Clinical evidence suggests that mirtazapine augmentation may accelerate or enhance SSRI response in OCD [30,31]. Additionally, through its 5-HT3 antagonism, mirtazapine may mitigate SSRI-related gastrointestinal intolerance such as nausea, potentially improving treatment adherence in patients sensitive to serotonergic side effects. In addition, mirtazapine-induced appetite stimulation may be advantageous in patients experiencing reduced appetite or weight loss associated with severe anxiety or serotonergic treatment. In the present case this pharmacological profile was considered particularly relevant given the coexistence of insomnia, anxiety, and persistent obsessive rumination. The third case demonstrates a dual SSRI strategy following inadequate response to optimized monotherapy. Evidence supporting the combination of two SSRIs remains limited and largely confined to case reports and observational experience [26,33]. In clinical practice, such strategies are occasionally considered in highly refractory situations where partial serotonergic responsiveness suggests that further modulation of the serotonergic system may overcome a therapeutic plateau. These approaches should however be reserved for carefully selected cases and implemented with close monitoring due to the potential risks of pharmacokinetic interactions, serotonergic toxicity, and QT prolongation. Dual SSRI strategies remain highly experimental and are not supported by randomized controlled trials. Given the limited evidence base and the potential risks associated with serotonergic polypharmacy, such strategies should be considered highly experimental and restricted to carefully selected treatment-refractory cases managed within specialist settings with structured safety monitoring. Their use should therefore be considered only in highly selected refractory cases and within specialist clinical settings with careful monitoring. From a circuit-based perspective, pharmacological intensification strategies may be conceptualized as attempts to restore functional balance within dysregulated cortico–striato–thalamo–cortical networks implicated in obsessive–compulsive symptom generation. The fourth case illustrates obsessive–compulsive symptoms occurring in the context of clozapine treatment for schizoaffective disorder. Clozapine-associated obsessive–compulsive symptoms represent a well-recognized clinical challenge, particularly because reduction in clozapine dosage may not be feasible due to the risk of psychotic relapse. In such cases, targeted pharmacological strategies aimed at modulating serotonergic pathways may represent a pragmatic clinical approach. Clozapine-associated obsessive–compulsive symptoms may occur in approximately 20–30% of clozapine-treated patients [46,47]. A recent scoping review confirms that obsessive–compulsive symptoms are common among clozapine-treated patients and remain a clinically important, under-recognized problem [48]. Targeted treatment of obsessive–compulsive symptoms with serotonergic pharmacotherapy may therefore represent a pragmatic clinical strategy when psychotic symptoms remain otherwise stable. When selecting serotonergic agents in patients receiving clozapine, metabolic considerations may also influence pharmacological choice. Clozapine treatment is frequently associated with significant weight gain and metabolic dysregulation. Among SSRIs, fluoxetine is generally considered relatively weight-neutral and has demonstrated efficacy in reducing binge-eating and bulimic behaviors. In patients treated with clozapine—such as the patient described in Case 4—where hyperphagia and metabolic burden may represent clinically relevant concerns, the use of fluoxetine may therefore represent a pragmatic pharmacological option.

These four serotonergic strategies are not equivalent in evidence, risk, or clinical acceptability, and should not be presented as interchangeable. High-dose SSRI optimization is the best-supported strategy, being grounded in dose–response data and a controlled high-dose sertraline trial and carries the most favorable risk profile. SSRI–mirtazapine augmentation has a modest but more direct evidence base, including a placebo-controlled trial, and is generally well tolerated. Serotonergic treatment of clozapine-associated obsessive–compulsive symptoms is a pragmatic, problem-specific strategy whose principal risk is pharmacokinetic (raised clozapine levels) rather than serotonergic. Dual SSRI therapy, by contrast, is the most experimental and highest-risk serotonergic strategy considered here: it is supported only by case reports and small observational series and combines additive serotonin toxicity and QT-prolongation liability. It should therefore be regarded as a last resort option, restricted to carefully selected refractory patients managed in specialist settings with structured safety monitoring.

Serotonergic intensification should also be positioned relative to third-generation dopamine partial-agonist antipsychotic augmentation, an emerging strategy for SRI-resistant OCD and directly relevant to the present series, as four patients had prior antipsychotic exposure and Case 5 included brexpiprazole in the background regimen. Recent retrospective studies have reported potential benefit with cariprazine and brexpiprazole augmentation in treatment-resistant OCD, providing a mechanistically distinct comparator to serotonergic intensification [49,50]. Similarly, preliminary evidence on multimodal serotonergic agents such as vortioxetine is relevant to Case 5, where vortioxetine formed part of the ongoing regimen. In patients in whom mood- or psychotic-spectrum instability complicates standard SSRI-based approaches, partial-agonist or long-acting antipsychotic strategies may be preferable to serotonergic polypharmacy [51,52]. Finally, the heterogeneity of dominant symptom dimensions across our cases supports a dimension-informed, personalized approach to treatment selection [53]. We therefore frame serotonergic intensification not as superior to antipsychotic augmentation, but as one of several mechanism-specific tools whose selection should be individualized.

### 5.2. Glutamatergic Augmentation

The final case illustrates augmentation with topiramate in a patient with persistent symptoms despite complex serotonergic pharmacotherapy. Increasing neurobiological evidence implicates glutamatergic dysregulation within cortico–striato–thalamo–cortical circuits in OCD pathophysiology [4]. This has led to growing interest in glutamate-modulating agents as potential treatments for refractory OCD. Topiramate enhances GABAergic transmission and inhibits AMPA/kainate glutamate receptors, potentially reducing pathological hyperactivity within frontostriatal circuits. The partial improvement observed with topiramate augmentation is consistent with emerging evidence implicating glutamatergic dysregulation within cortico–striato–thalamo–cortical circuits in treatment-resistant OCD. Randomized controlled trials have reported reductions in compulsive symptoms when topiramate is added to ongoing SSRI therapy in treatment-resistant OCD populations [34,35]. From a clinical perspective, topiramate may be particularly attractive in patients with treatment-resistant OCD who also experience weight gain related to psychotropic medications or impulsive-compulsive eating behaviors. Topiramate has demonstrated efficacy in binge-eating disorder and has been associated with reductions in binge frequency and body weight in randomized trials [54]. The pharmacological regimen observed in Case 5 reflects a multi-level neurobiological strategy targeting serotonergic, dopaminergic, and glutamatergic systems implicated in cortico–striato–thalamo–cortical circuit dysfunction. However, tolerability limitations—including cognitive slowing, paresthesia, and fatigue—may restrict broader use and require careful monitoring. These considerations argue against presenting topiramate as a broadly attractive option. In the present series it produced only partial improvement (23% Y-BOCS reduction), and its weight-related and binge-eating advantages were not applicable to our patient, who had no such features. We therefore regard topiramate augmentation as a cautious, patient-specific choice—reasonable chiefly when a glutamatergic mechanism is sought and/or when comorbid weight gain, binge eating, or impulsivity provides an additional, individual-level rationale—rather than a generally recommended strategy.

### 5.3. Safety Considerations

Safety considerations are central when employing serotonergic polypharmacy or high-dose pharmacotherapy. Potential risks include serotonin syndrome, pharmacokinetic drug–drug interactions mediated by cytochrome P450 enzymes, QT interval prolongation, and reductions in seizure threshold. In the present case series, pharmacological intensification strategies were implemented using gradual titration and structured monitoring protocols, including electrocardiographic evaluation when clinically indicated. No cases of serotonin syndrome or clinically significant cardiac complications were observed. These cases also highlight the importance of structured safety monitoring when pharmacological intensification strategies are considered, including electrocardiographic assessment, careful dose titration, and systematic evaluation of serotonin toxicity. Taken together, these observations suggest that pharmacological intensification strategies may represent pragmatic options in highly treatment-resistant OCD when guideline-supported interventions have failed. However, such approaches should be implemented cautiously and ideally within specialist clinical settings with careful monitoring and individualized risk–benefit assessment.

## 6. Limitations

Several limitations should be acknowledged. First, the present study includes a small observational case series without a control group, which limits the ability to draw causal conclusions regarding treatment efficacy. The clinical improvements observed may therefore reflect multiple factors, including natural fluctuations in symptom severity or concurrent non-pharmacological interventions. Second, the pharmacological strategies described were heterogeneous, reflecting individualized clinical decision-making in a tertiary referral setting. While this heterogeneity mirrors real-world practice, it prevents direct comparison between interventions and limits the ability to identify which specific strategy may be most effective for particular patient subgroups. Third, the cases were collected in a specialized outpatient clinic for treatment-resistant OCD. As a result, the clinical characteristics of the patients and the therapeutic approaches implemented may not be fully generalizable to broader psychiatric populations or general outpatient settings. Finally, although the present report highlights potential clinical scenarios in which pharmacological intensification strategies may be considered, the observations presented here should be interpreted as hypothesis-generating rather than definitive evidence. Controlled studies are required to clarify the efficacy, safety, and optimal positioning of these strategies within treatment algorithms for treatment-resistant OCD. The small number of cases reflects the highly selected nature of patients referred to a tertiary treatment-resistant OCD service and the exploratory objective of illustrating real-world clinical decision-making rather than establishing treatment efficacy.

Several further, more specific limitations warrant emphasis. The denominator of eligible patients screened during the study period is not fully reconstructed, so selection bias cannot be excluded. Week-12 Y-BOCS outcomes were obtained retrospectively from the records, ratings were performed by treating (non-blinded) clinicians rather than independent assessors, and outcomes were not adjudicated; together these factors may inflate apparent improvement. In the absence of a control group, the observed changes are also vulnerable to regression to the mean and to natural symptom fluctuation. Follow-up was short (12 weeks), precluding any inference about durability, and the interventions and concurrent background treatments were heterogeneous, preventing attribution of change to any single agent. ERP adequacy was documented only narratively and was incomplete or unavailable in several cases, which weakens the “treatment-resistant” label. Finally, outcomes relied almost exclusively on the Y-BOCS, without standardized functional or patient-reported measures. For all these reasons, and given a sample of only five patients, no statistically or clinically generalizable conclusion about efficacy can be drawn; the series is strictly hypothesis-generating.

## 7. Recommendations

On the basis of this experience and the synthesized evidence, we offer the following practical recommendations for clinicians managing highly refractory OCD. First, pharmacological intensification beyond guideline algorithms should be considered only after genuine optimization of SRI/clomipramine dosing and duration and a documented attempt at exposure and response prevention, and should be reserved for specialist settings. Second, before adding agents, clinicians should confirm that apparent resistance is not in fact incomplete serotonergic exposure—high-dose SSRI optimization is the best-supported, lowest-risk first step. Third, when combination strategies are used, they should be introduced one agent at a time with gradual titration, structured screening for serotonin toxicity (e.g., Hunter criteria), and baseline and follow-up QTcF measurement, with predefined thresholds for re-evaluation and discontinuation. Fourth, dual SSRI therapy should be treated as a last-resort, highest-risk option and avoided where safer alternatives (antipsychotic or third-generation antipsychotic augmentation, mirtazapine augmentation, glutamatergic agents, or neuromodulation) are available. Fifth, treatment selection should be individualized and dimension-informed, taking account of comorbidity (e.g., tic history, psychosis, metabolic burden) and patient preference, and decisions should be shared and documented within a multidisciplinary, risk–benefit framework. Finally, given the weakness of the current evidence base, these strategies should ideally be delivered within prospective registries or controlled studies that incorporate standardized functional and patient-reported outcomes.

## 8. Conclusions

Treatment-resistant obsessive–compulsive disorder remains a major therapeutic challenge. Even after optimized SSRI therapy, clomipramine trials, antipsychotic augmentation, and behavioral interventions, a subset of patients continues to experience severe and functionally impairing symptoms. The present case series illustrates how pharmacological intensification strategies—including supratherapeutic SSRI dosing, serotonergic combination therapy, and glutamatergic augmentation—may be feasible and offer possible benefit in selected patients managed within specialized clinical settings. Importantly, the cases presented suggest that in some individuals treatment resistance may reflect incomplete rather than absent serotonergic responsiveness, supporting cautious exploration of intensification strategies before moving toward more invasive or device-based interventions. Although preliminary, these observations underscore the importance of individualized, mechanism-informed pharmacological decision-making in highly refractory OCD. Further controlled studies are needed to better define the clinical profiles most likely to benefit from these approaches and to establish their safety and efficacy within contemporary treatment frameworks. Overall, these cases suggest that carefully monitored pharmacological intensification may be feasible in selected specialist settings, but efficacy and safety require confirmation in prospective controlled studies.

## Figures and Tables

**Table 1:** Clinical characteristics, background pharmacotherapy, intensification strategy, and outcomes.

Case	Age/Sex	Comorbidity	Background Regimen	Intensification Strategy	Baseline Y-BOCS	Baseline CGI-S	Y-BOCS (12 wk)	CGI-I (12 wk)	Outcome
1	62 F	None reported	Sertraline 200 mg/day + Risperidone 2 mg/day + Lorazepam 1 mg at bedtime	Sertraline increased to 250 mg/day	28	6	18	2	Response (36%)
2	59 F	Generalized anxiety, insomnia	Fluoxetine 60 mg/day + Pregabalin	Mirtazapine added (45 mg/day)	29	6	19	2	Near-response (34%)
3	47 M	Motor tics	Fluoxetine 80 mg/day	Switch to Sertraline 200 mg/day + Citalopram 40 mg/day	32	7	21	2	Near-response (34%)
4	33 M	Schizoaffective disorder	Clozapine 200 mg/day + Valproate 500 mg BID	Sertraline 200 mg/day + Fluoxetine 40 mg/day added	31	6	21	2	Near-response (32%)
5	38 M	None reported	Paroxetine 60 mg/day + Vortioxetine 20 mg/day + Brexpiprazole 2 mg/day	Topiramate added (100 mg/day)	30	6	23	3	Partial response (23%)

Note: Response, ≥35% Y-BOCS reduction; near-response, 25–34%; partial response, <25%. CGI-S, Clinical Global Impression-Severity (1 = normal to 7 = among the most extremely ill); CGI-I, Clinical Global Impression-Improvement (1 = very much improved, 2 = much improved, 3 = minimally improved, 4 = no change, 5–7 = worsening).

**Table 2:** Treatment history and safety-monitoring details for the case series.

Case	Illness Duration	ERP Status	Prior SRI Trials (Incl. Clomipramine)	Prior Antipsychotic Augmentation	ECG/QTcF	Main Adverse Effects/Dose Stability
1 (62 F)	Onset in early adulthood; episodic (~40 yrs)	Refused	Fluvoxamine, citalopram, clomipramine, sertraline (≥4; clomipramine yes)	Aripiprazole (d/c, no benefit); risperidone 2 mg (ongoing)	Normal at baseline and wk 12; no QTcF prolongation (<480 ms)	None; only sertraline up-titrated (200 → 250 mg), other doses stable
2 (59 F)	Onset at age 20 (~39 yrs)	Ongoing	Multiple SRIs incl. fluoxetine 60 mg; clomipramine yes	Aripiprazole (akathisia); risperidone (hyperprolactinaemia)	Normal at baseline and wk 12; no QTcF prolongation (<480 ms)	Transient sedation; fluoxetine 60 mg stable, mirtazapine added to 45 mg
3 (47 M)	Since childhood; tic history (~35 yrs)	Ongoing	Fluoxetine 80 mg, fluvoxamine 300 mg; clomipramine yes	Aripiprazole 10 mg; risperidone 2 mg (no benefit)	Normal at baseline and wk 12; no QTcF prolongation (<480 ms)	None; dual SSRI doses stable after titration
4 (33 M)	Since adolescence; schizoaffective comorbidity (~20 yrs)	Ongoing	Sertraline 200 mg, fluoxetine 40 mg; clomipramine yes	Clozapine 200 mg/day (psychosis) + valproate; clozapine 157/norclozapine 75 ng/mL	Normal at baseline and wk 12; no QTcF prolongation (<480 ms)	None; clozapine/valproate held constant, no psychotic relapse
5 (38 M)	Since adolescence (~25 yrs)	Ongoing	Multiple SRIs (paroxetine 60 mg, vortioxetine 20 mg); clomipramine yes	Prior SGAs; current brexpiprazole 2 mg	Normal at baseline and wk 12; no QTcF prolongation (<480 ms)	Transient paresthesia; background regimen stable, topiramate added to 100 mg

Abbreviations: ERP, exposure and response prevention; SRI, serotonin reuptake inhibitor; SGA, second-generation antipsychotic; QTcF, Fridericia-corrected QT interval; d/c, discontinued. Illness durations for Cases 1, 3, 4 and 5 are approximate, derived from documented age at onset.

## Data Availability

The data underlying the case series are not publicly available due to privacy and ethical restrictions. Further information may be available from the corresponding author upon reasonable request and in accordance with applicable regulations.

## References

[1] Stein D.J., Costa D.L.C., Lochner C., Miguel E.C., Reddy Y.C.J., Shavitt R.G., van den Heuvel O.A., Simpson H.B. (2019). Obsessive-compulsive disorder. Nat. Rev. Dis. Primers.

[2] Fontenelle I.S., Fontenelle L.F., Borges M.C., Prazeres A.M., Rangé B.P., Mendlowicz M.V., Versiani M. (2010). Quality of life and symptom dimensions of patients with obsessive-compulsive disorder. Psychiatry Res..

[3] Dos Santos-Ribeiro S., de Menezes G.B., Moreira-de-Oliveira M.E., Hühne V., Fortes P.P., Fontenelle L.F. (2025). The effect of treatment on the quality of life of patients with obsessive-compulsive disorder: Systematic review and meta-analysis. J. Psychiatr. Res..

[4] Pittenger C., Bloch M.H., Williams K. (2011). Glutamate abnormalities in obsessive compulsive disorder: Neurobiology, pathophysiology, and treatment. Pharmacol. Ther..

[5] Koran L.M., Hanna G.L., Hollander E., Nestadt G., Simpson H.B. (2007). Practice guideline for the treatment of patients with obsessive-compulsive disorder. Am. J. Psychiatry.

[6] National Institute for Health and Care Excellence (2005). Obsessive-Compulsive Disorder and Body Dysmorphic Disorder: Treatment.

[7] Skapinakis P., Caldwell D.M., Hollingworth W., Bryden P., Fineberg N.A., Salkovskis P., Welton N.J., Baxter H., Kessler D., Churchill R. (2016). Pharmacological and psychotherapeutic interventions for management of obsessive-compulsive disorder in adults: A systematic review and network meta-analysis. Lancet Psychiatry.

[8] Quercioli L., Pallanti S. (2006). Treatment-refractory obsessive-compulsive disorder: Methodological issues, operational definitions and therapeutic lines. Prog. Neuropsychopharmacol. Biol. Psychiatry.

[9] Carmi L., Tendler A., Bystritsky A., Hollander E., Blumberger D.M., Daskalakis J., Ward H., Lapidus K., Goodman W., Casuto L. (2019). Efficacy and Safety of Deep Transcranial Magnetic Stimulation for Obsessive-Compulsive Disorder: A Prospective Multicenter Randomized Double-Blind Placebo-Controlled Trial. Am. J. Psychiatry.

[10] Denys D., Mantione M., Figee M., van den Munckhof P., Koerselman F., Westenberg H., Bosch A., Schuurman R. (2010). Deep brain stimulation of the nucleus accumbens for treatment-refractory obsessive-compulsive disorder. Arch. Gen. Psychiatry.

[11] van Roessel P.J., Grassi G., Aboujaoude E.N., Menchón J.M., Van Ameringen M., Rodríguez C.I. (2023). Treatment-resistant OCD: Pharmacotherapies in adults. Compr. Psychiatry.

[12] von Elm E., Altman D.G., Egger M., Pocock S.J., Gøtzsche P.C., Vandenbroucke J.P. (2007). The Strengthening the Reporting of Observational Studies in Epidemiology (STROBE) statement: Guidelines for reporting observational studies. Lancet.

[13] Gagnier J.J., Kienle G., Altman D.G., Moher D., Sox H., Riley D. (2013). The CARE guidelines: Consensus-based clinical case reporting guideline development. Headache.

[14] Goodman W.K., Price L.H., Rasmussen S.A., Mazure C., Fleischmann R.L., Hill C.L., Heninger G.R., Charney D.S. (1989). The Yale-Brown Obsessive Compulsive Scale: I. Development, Use, and Reliability. Arch. Gen. Psychiatry.

[15] Dunkley E.J., Isbister G.K., Sibbritt D., Dawson A.H., Whyte I.M. (2003). The Hunter Serotonin Toxicity Criteria: Simple and accurate diagnostic decision rules for serotonin toxicity. QJM.

[16] Menzies L., Chamberlain S.R., Laird A.R., Thelen S.M., Sahakian B.J., Bullmore E.T. (2008). Integrating evidence from neuroimaging and neuropsychological studies of obsessive-compulsive disorder: The orbitofronto-striatal model revisited. Neurosci. Biobehav. Rev..

[17] Milad M.R., Rauch S.L. (2012). Obsessive-compulsive disorder: Beyond segregated cortico-striatal pathways. Trends Cogn. Sci..

[18] Rotge J.Y., Guehl D., Dilharreguy B., Tignol J., Bioulac B., Allard M., Burbaud P., Aouizerate B. (2009). Meta-analysis of brain volume changes in obsessive-compulsive disorder. Biol. Psychiatry.

[19] Fajnerova I., Gregus D., Francova A., Noskova E., Koprivova J., Stopkova P., Hlinka J., Horacek J. (2020). Functional Connectivity Changes in Obsessive-Compulsive Disorder Correspond to Interference Control and Obsessions Severity. Front. Neurol..

[20] Perera M.P.N., Gotsis E.S., Bailey N.W., Fitzgibbon B.M., Fitzgerald P.B. (2024). Exploring functional connectivity in large-scale brain networks in obsessive-compulsive disorder: A systematic review of EEG and fMRI studies. Cereb. Cortex..

[21] Bandelow B., Allgulander C., Baldwin D.S., Costa D., Denys D., Dilbaz N., Domschke K., Hollander E., Kasper S., Möller H.J. (2023). World Federation of Societies of Biological Psychiatry (WFSBP) guidelines for treatment of anxiety, obsessive-compulsive and posttraumatic stress disorders–Version 3. Part II: OCD and PTSD. World J. Biol. Psychiatry.

[22] Bloch M.H., Landeros-Weisenberger A., Kelmendi B., Coric V., Bracken M.B., Leckman J.F. (2006). A systematic review: Antipsychotic augmentation with treatment refractory obsessive-compulsive disorder. Mol. Psychiatry.

[23] Dold M., Aigner M., Lanzenberger R., Kasper S. (2015). Antipsychotic Augmentation of Serotonin Reuptake Inhibitors in Treatment-Resistant Obsessive-Compulsive Disorder: An Update Meta-Analysis of Double-Blind, Randomized, Placebo-Controlled Trials. Int. J. Neuropsychopharmacol..

[24] Suhas S., Malo P.K., Kumar V., Issac T.G., Chithra N.K., Bhaskarapillai B., Reddy Y.C.J., Rao N.P. (2023). Treatment strategies for serotonin reuptake inhibitor-resistant obsessive-compulsive disorder: A network meta-analysis of randomised controlled trials. World J. Biol. Psychiatry.

[25] Maiti R., Mishra A., Srinivasan A., Mishra B.R. (2023). Pharmacological augmentation of serotonin reuptake inhibitors in patients with obsessive-compulsive disorder: A network meta-analysis. Acta. Psychiatr. Scand..

[26] Kayser R.R. (2020). Pharmacotherapy for Treatment-Resistant Obsessive-Compulsive Disorder. J. Clin. Psychiatry.

[27] Bloch M.H., McGuire J., Landeros-Weisenberger A., Leckman J.F., Pittenger C. (2010). Meta-analysis of the dose-response relationship of SSRI in obsessive-compulsive disorder. Mol. Psychiatry.

[28] Ninan P.T., Koran L.M., Kiev A., Davidson J.R., Rasmussen S.A., Zajecka J.M., Robinson D.G., Crits-Christoph P., Mandel F.S., Austin C. (2006). High-dose sertraline strategy for nonresponders to acute treatment for obsessive-compulsive disorder: A multicenter double-blind trial. J. Clin. Psychiatry.

[29] Levy D.M., Arush O.B., Carmi L., Wetzler A.J., Zohar J. (2024). Off-label higher doses of serotonin reuptake inhibitors in the treatment of obsessive-compulsive disorder: Safety and tolerability. Compr. Psychiatry.

[30] Koran L.M., Gamel N.N., Choung H.W., Smith E.H., Aboujaoude E.N. (2005). Mirtazapine for obsessive-compulsive disorder: An open trial followed by double-blind discontinuation. J. Clin. Psychiatry.

[31] Mowla A., Baniasadipour H. (2023). Is mirtazapine augmentation effective for patients with obsessive-compulsive disorder who failed to respond to sertraline monotherapy? A placebo-controlled, double-blind, clinical trial. Int. Clin. Psychopharmacol..

[32] Andrade C. (2013). Augmenting selective serotonin reuptake inhibitors with clomipramine in obsessive-compulsive disorder: Benefits and risks. J. Clin. Psychiatry.

[33] Arumugham S.S., Reddy J.Y. (2013). Augmentation strategies in obsessive-compulsive disorder. Expert Rev. Neurother..

[34] Mowla A., Khajeian A.M., Sahraian A., Chohedri A.H., Kashkoli F. (2010). Topiramate Augmentation in Resistant OCD: A Double-Blind Placebo-Controlled Clinical Trial. CNS Spectr..

[35] Berlin H.A., Koran L.M., Jenike M.A., Shapira N.A., Chaplin W., Pallanti S., Hollander E. (2011). Double-blind, placebo-controlled trial of topiramate augmentation in treatment-resistant obsessive-compulsive disorder. J. Clin. Psychiatry.

[36] Costa D.L.C., Diniz J.B., Requena G., Joaquim M.A., Pittenger C., Bloch M.H., Miguel E.C., Shavitt R.G. (2017). Randomized, Double-Blind, Placebo-Controlled Trial of N-Acetylcysteine Augmentation for Treatment-Resistant Obsessive-Compulsive Disorder. J. Clin. Psychiatry.

[37] Afshar H., Roohafza H., Mohammad-Beigi H., Haghighi M., Jahangard L., Shokouh P., Sadeghi M., Hafezian H. (2012). N-acetylcysteine add-on treatment in refractory obsessive-compulsive disorder: A randomized, double-blind, placebo-controlled trial. J. Clin. Psychopharmacol..

[38] Eghdami S., Eissazade N., Heidari Mokarar M., Boroon M., Orsolini L., Shalbafan M. (2024). The safety and efficacy of N-acetylcysteine as an augmentation in the treatment of obsessive-compulsive disorder in adults: A systematic review and meta-analysis of randomized clinical trials. Front. Psychiatry.

[39] Zhang W., Zhang Y., Yu E. (2025). Effectiveness and Safety of Memantine Add-on Treatment for Refractory Obsessive-Compulsive Disorder: A Meta-Analysis. J. Coll. Physicians Surg. Pak..

[40] Rodriguez C.I., Kegeles L.S., Levinson A., Feng T., Marcus S.M., Vermes D., Flood P., Simpson H.B. (2013). Randomized controlled crossover trial of ketamine in obsessive-compulsive disorder: Proof-of-concept. Neuropsychopharmacology.

[41] Beaglehole B., Glue P., Neehoff S., Shadli S., McNaughton N., Kimber B., Muirhead C., Bie A., Day-Brown R., Hughes-Medlicott N.J. (2025). Ketamine for treatment-resistant obsessive-compulsive disorder: Double-blind active-controlled crossover study. J. Psychopharmacol..

[42] Bandeira I.D., Lins-Silva D.H., Cavenaghi V.B., Dorea-Bandeira I., Faria-Guimarães D., Barouh J.L., Jesus-Nunes A.P., Beanes G., Souza L.S., Leal G.C. (2022). Ketamine in the Treatment of Obsessive-Compulsive Disorder: A Systematic Review. Harv. Rev. Psychiatry.

[43] Cruz S., Gutiérrez-Rojas L., González-Domenech P., Díaz-Atienza F., Martínez-Ortega J.M., Jiménez-Fernández S. (2022). Deep brain stimulation in obsessive-compulsive disorder: Results from meta-analysis. Psychiatry Res..

[44] Li K., Qian L., Zhang C., Li R., Zeng J., Xue C., Deng W. (2024). Deep transcranial magnetic stimulation for treatment-resistant obsessive-compulsive disorder: A meta-analysis of randomized-controlled trials. J. Psychiatr. Res..

[45] McDougle C.J., Goodman W.K., Leckman J.F., Lee N.C., Heninger G.R., Price L.H. (1994). Haloperidol addition in fluvoxamine-refractory obsessive-compulsive disorder. A double-blind, placebo-controlled study in patients with and without tics. Arch. Gen. Psychiatry.

[46] Schirmbeck F., Zink M. (2012). Clozapine-induced obsessive-compulsive symptoms in schizophrenia: A critical review. Curr. Neuropharmacol..

[47] Fonseka T.M., Richter M.A., Müller D.J. (2014). Second generation antipsychotic-induced obsessive-compulsive symptoms in schizophrenia: A review of the experimental literature. Curr. Psychiatry Rep..

[48] Moreno Tarazona E., Orozco Gonzalez M., La Rosa Giron A., Ruiz-Grosso P., Lazo-Porras M. (2025). Prevalence of obsessive-compulsive symptoms in patients with schizophrenia treated with clozapine: A scoping review. BMC Psychiatry.

[49] Martiadis V., Pessina E., Martini A., Raffone F., Cattaneo C.I., De Berardis D., Pampaloni I. (2024). Serotonin reuptake inhibitors augmentation with cariprazine in patients with treatment-resistant obsessive-compulsive disorder: A retrospective observational study. CNS Spectr..

[50] Martiadis V., Pessina E., Martini A., Raffone F., Besana F., Olivola M., Cattaneo C.I. (2024). Brexpiprazole Augmentation in Treatment Resistant OCD: Safety and Efficacy in an Italian Sample. Psychiatr. Danub..

[51] Martiadis V., Pessina E., Cattaneo C.I., Martini A., Raffone F., Prodi T., Olivola M., De Berardis D., Benatti B., Dell’Osso B.M. (2025). Efficacy and tolerability of vortioxetine monotherapy in SSRI-resistant OCD: A retrospective multicenter study. Front. Psychiatry.

[52] Martiadis V., Pessina E., Raffone F., Martini A., Di Vincenzo M., Della Rocca B., De Berardis D., Cattaneo C.I., Sampogna G. (2025). Efficacy and Safety of Adjunctive Aripiprazole LAI or Paliperidone LAI for the Management of Patients Suffering from Bipolar I Disorder with Comorbid Obsessive-Compulsive Disorder. J. Clin. Med..

[53] Martiadis V., Raffone F., Iaccarino C., Carbone E., De Simone C., Purcaro C., Olivola M., Barlattani T., De Berardis D., Pacitti F. (2026). Metacognitive Belief Profiles Across OCD Symptom Dimensions: A Systematic Review and Clinical Implications for Personalised Treatment. J. Clin. Med..

[54] McElroy S.L., Hudson J.I., Capece J.A., Beyers K., Fisher A.C., Rosenthal N.R. (2007). Topiramate for the treatment of binge eating disorder associated with obesity: A placebo-controlled study. Biol. Psychiatry.

